# Robotic posterior retroperitoneal adrenalectomy versus laparoscopic posterior retroperitoneal adrenalectomy: outcomes from a pooled analysis

**DOI:** 10.3389/fendo.2023.1278007

**Published:** 2023-11-28

**Authors:** Yu-gen Li, Xiao-bin Chen, Chun-mei Wang, Xiao-dong Yu, Xian-zhong Deng, Bo Liao

**Affiliations:** ^1^ Department of Urology, Affiliated Hospital of North Sichuan Medical College, Nan chong, China; ^2^ Physical Examination Center, Affiliated Hospital of North Sichuan Medical College, Nan chong, China

**Keywords:** robotic, laparoscopic, posterior, adrenalectomy, meta-analysis

## Abstract

**Background:**

The comparative advantages of robotic posterior retroperitoneal adrenalectomy (RPRA) over laparoscopic posterior retroperitoneal adrenalectomy (LPRA) remain a topic of ongoing debate within the medical community. This systematic literature review and meta-analysis aim to assess the safety and efficacy of RPRA compared to LPRA, with the ultimate goal of determining which procedure yields superior clinical outcomes.

**Methods:**

A systematic search was conducted on databases including PubMed, Embase, Web of Science, and the Cochrane Library database to identify relevant studies, encompassing both randomized controlled trials (RCTs) and non-RCTs, that compare the outcomes of RPRA and LPRA. The primary focus of this study was to evaluate perioperative surgical outcomes and complications. Review Manager 5.4 was used for this analysis. The study was registered with PROSPERO (ID: CRD42023453816).

**Results:**

A total of seven non-RCTs were identified and included in this study, encompassing a cohort of 675 patients. The findings indicate that RPRA exhibited superior performance compared to LPRA in terms of hospital stay (weighted mean difference [WMD] -0.78 days, 95% confidence interval [CI] -1.46 to -0.10; p = 0.02). However, there were no statistically significant differences observed between the two techniques in terms of operative time, blood loss, transfusion rates, conversion rates, major complications, and overall complications.

**Conclusion:**

RPRA is associated with a significantly shorter hospital stay compared to LPRA, while demonstrating comparable operative time, blood loss, conversion rate, and complication rate. However, it is important to note that further research of a more comprehensive and rigorous nature is necessary to validate these findings.

**Systematic review registration:**

https://www.crd.york.ac.uk/prospero/display_record.php?RecordID=453816, identifier CRD42023453816.

## 1 Introduction

Laparoscopic transperitoneal adrenalectomy (LTA) was first elucidated by Gagner et al. in 1992 (1). Subsequent empirical evidence has unequivocally unveiled a spectrum of advantages inherently associated with LTA, transcending those of conventional open adrenalectomy. These encompass a notable reduction in estimated blood loss, abbreviated hospitalization periods, alleviated postoperative discomfort, and a diminished incidence of complications (2, 3). In the year 1995, Mercan et al. introduced the laparoscopic posterior retroperitoneal adrenalectomy (LPRA), an innovative surgical paradigm that has since been methodically established as both a practicable and secure operative approach (4–6). From an anatomical vantage point, LPRA presents a more direct conduit to reach the adrenal gland, obviating the necessity for the mobilization of contiguous structures. This tactical approach concomitantly mitigates the potential for complications entailed in peritoneal cavity ingress. Noteworthy is LPRA’s specific commendation for patients harboring bilateral tumors and grappling with abdominal adhesions (7). However, juxtaposed against LTA, LPRA does encounter certain limitations stemming from its confined surgical arena, rigid instrumentation, and plausible interactions with neighboring anatomical architecture (8).

In the realm of surgical innovation, propelled by advancements in technology, robotic posterior retroperitoneal adrenalectomy (RPRA) has ascended as a preeminent surgical modality. RPRA affords an array of advantages, including heightened visual acuity through three-dimensional optics and an expanded panoramic canvas of the operative field. This is coupled with a broader range of maneuverability, encompassing seven degrees of freedom compared to the conventional four, thereby enhancing the ergonomic milieu for surgical practitioners (9, 10). However, it is imperative to acknowledge the attendant limitations inherent in robotic surgical systems, encompassing the requisites of setup, the intricacies of instrumentation, augmented expenses, and an extended surgical duration. As a result, the quest for the optimal surgical paradigm within the constricted confines of the retroperitoneal space remains a matter of ongoing scholarly discourse.

Therefore, the aim of this study is to amalgamate data derived from comparative studies and evaluate the efficacy and safety of RPRA and LPRA. The results of this study are intended to function as an all-encompassing guide for clinical decision-making, thereby assisting physicians in the discernment of the most fitting surgical approach for their patients.

## 2 Methods

This study was executed in strict adherence to the protocols delineated within the Preferred Reporting Items for Systematic Reviews and Meta-Analyses (PRISMA) statement [13]. Furthermore, it underwent registration within the PROSPERO database (ID: CRD42023453816) in accordance with established practices.

### 2.1 Literature search strategy, study selection and data collection

A thorough and exhaustive electronic survey of the academic databases, encompassing PubMed, Embase, Web of Science, and the Cochrane Library, was meticulously conducted. The data collection process was finalized in July 2023. The search strategy seamlessly integrated pertinent terms concerning the intervention and patient demographics, culminating in the formulation of the subsequent search query: ((Laparoscopic OR Robot-assisted OR Minimally invasive) AND (Retroperitoneoscopic OR Retroperitoneal OR Direct posterior OR Posterior) AND (Adrenalectomy)). Additionally, a comprehensive manual inquiry and scrupulous assessment of pertinent studies were undertaken to ensure the preemptive mitigation of potential oversights. It is noteworthy that the search was specifically delimited to publications presented in the English language. In instances of discordance, a consensus was judiciously attained through deliberation or, when deemed necessary, through consultation with a third reviewer.

The criteria for inclusion were delineated utilizing the PICOS methodology. P (patients): Patients aged 18 years or older who were due to undergo adrenalectomy for any indication. I (intervention): Encompassing patients subjected to RPRA. C (comparator): LPRA was employed as the comparative modality. O (outcome): The primary objective of this inquiry was to evaluate one or more of the ensuing outcomes: perioperative ramifications, surgical outcomes, and associated complications. S (study type): This investigation encompassed randomized controlled trials (RCTs), alongside both retrospective and prospective comparative analyses. Exclusionary criteria were employed as follows: (1) Studies bereft of comparative designs were systematically excluded. (2) Editorial commentaries, epistolary exchanges with the editor, abstracts from meetings, and singular case reports were not integrated into the analytical framework. (3) Studies that did not undertake an evaluation of the stipulated outcome metrics were purposefully excluded from consideration.

Following that, two independent reviewers meticulously extracted the subsequent dataset from the incorporated studies: (1) General manuscript details encompassing the year of publication, lead author, and country of origin. (2) Characteristics of the study population including sample size, age distribution, and body mass index (BMI). (3) Attributes specific to the tumors under investigation: tumor diameter, tumor site, and oncologic outcomes. (4) Perioperative effectiveness metrics: procedural duration, volume of blood loss, duration of hospitalization, rate of conversions, and frequency of transfusions. (5) Considerations regarding perioperative safety: overall complications (as defined by Clavien grade ≥ 1) and major complications (as defined by Clavien grade ≥ 3) (11). The process of data extraction was autonomously executed by the two reviewers to ensure meticulousness and uniformity.

In order to assess the quality of the literature, a comprehensive evaluation was conducted on the studies incorporated in the analysis, employing the “risk of bias in non-randomized studies of interventions” (ROBINS-I) framework (12). This assessment was executed independently by two evaluators (Y.L. and X.C.), who conducted a meticulous scrutiny of the studies for potential biases, encompassing confounding factors or other potential sources of systematic deviation. Any inconsistencies or differences that emerged during the assessment process were resolved through in-depth discourse.

### 2.2 Statistical analysis

For the purpose of data analysis within this study, we used the Cochrane Collaborative RevMan 5.4 software. Odds ratios (OR) were applied to assess dichotomous outcomes, while weighted mean differences (WMD) were employed to quantify continuous outcomes, accompanied by 95% confidence intervals (CI) for all evaluated measures. The evaluation of inter-study heterogeneity was accomplished using the I^2^ test (13). Given the anticipated existence of inter-trial heterogeneity, we adopted the random-effects model for all analyses, and statistical significance was determined at a significance threshold of p < 0.05. In instances where substantial heterogeneity was observed among outcomes (I^2^ > 75%), sensitivity analyses were undertaken to ascertain the origin of inter-study variability and to verify the robustness of our findings. However, sensitivity analyses could not be conducted for outcomes predicated on three or fewer studies.

### 2.3 Subgroup analysis

We performed a subgroup analysis considering several factors, such as age, BMI, sample size, and tumor diameter.

### 2.4 Publication bias

To evaluate the possibility of publication bias, we employed Begg’s method funnel plot.

## 3 Results

### 3.1 Baseline characteristics

**Figure 1:**
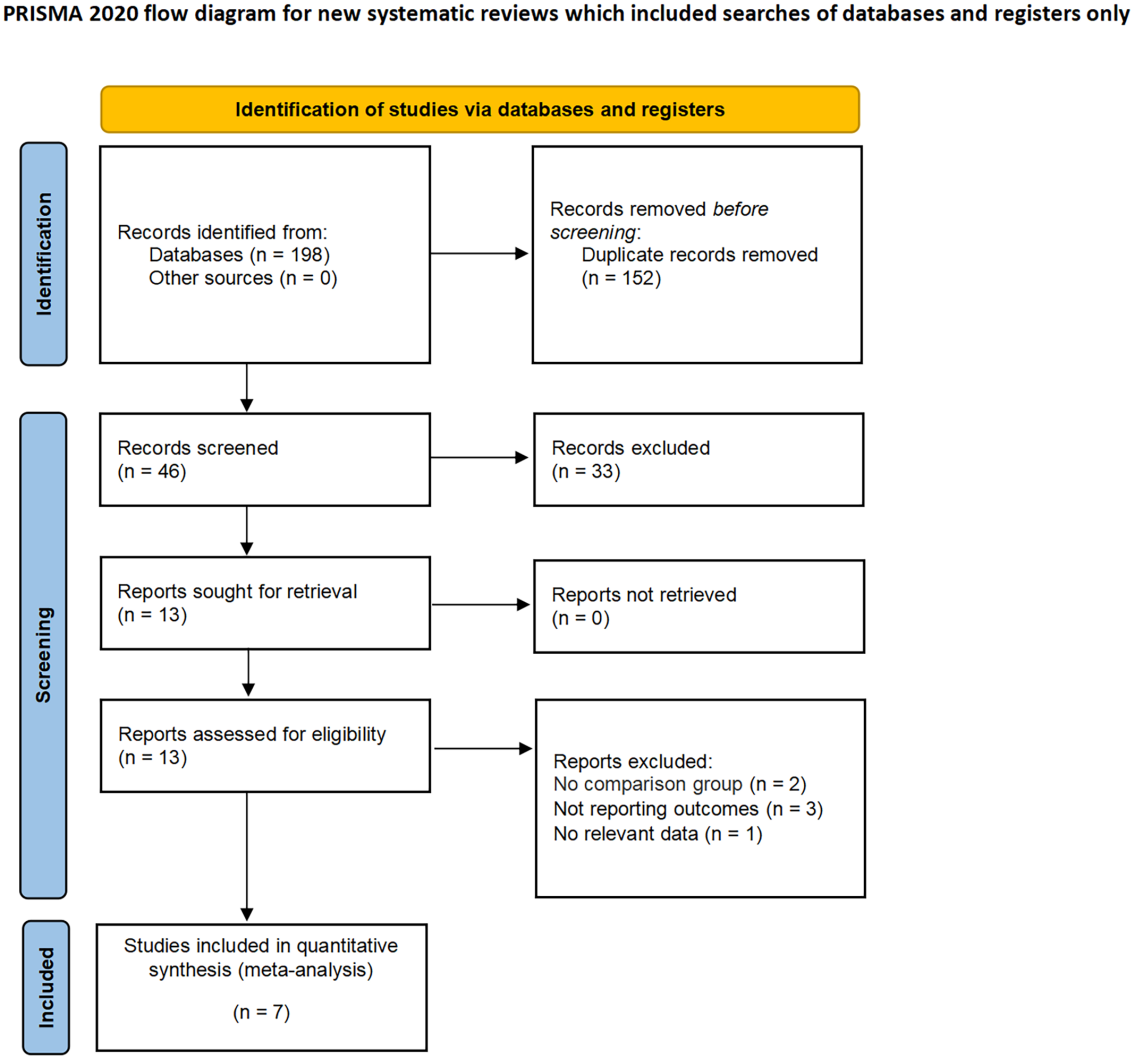
PRISMA flow diagram for the systematic review.

The applied search algorithm initially identified a total of 139 studies within the databases. Following an extensive review of full-text materials and a meticulous screening process, seven studies, comprising 675 patients in total, were deemed suitable for inclusion in the comprehensive meta-analysis (**Figure 1**) (14–20). All seven investigations adopted the posterior retroperitoneal adrenalectomy approach. The succinct overview in **Table 1** offers a synopsis of fundamental patient characteristics, accompanied by the corresponding interventions and associated preoperative variables (including sample size, age, BMI, surgical approach, tumor diameter, and location). These studies were conducted across diverse countries, including China, the United States, and Korea, and were published between 2012 and 2023. **Table 2** delineates perioperative and surgical outcomes, encompassing pivotal parameters such as operative duration, blood loss, hospitalization duration, conversion rate, transfusion frequency, and occurrences of complications. The compendious summation of oncologic outcomes is presented in **Table 3**.

**Table 1:** The trials included in the systemic review.

Reference	Year	Country	Age(y)		BMI (kg/m^2^)		Patients		Tumor diameter(cm)		Tumor site (Lt / Rt)		Surgical approach
			RPRA	LPRA	RPRA	LPRA	RPRA	LPRA	RPRA	LPRA	RPRA	LPRA	
Isiktas	2023	USA	53.9(19.3)	51.6(10.22)	37.9(2)	38.2(2.74)	15	24	3.58(1.63)	2.80(2.19)	06-Sep	18/6	Retroperitoneal
Ma	2021	China	45(14.82)	49(13.33)	23.53(3.43)	23.62(3.22)	79	79	3.5(1.63)	3.2(1.48)	47/32	46/33	Retroperitoneal
Fu	2020	China	44(9.062)	47.53(14.048)	26.64(3.82)	25.84(4.45)	19	32	8(2.22)	7.65(1.76)	10-Sep	16/16	Retroperitoneal
Kim	2019	Korea	46.5(11.6)	50.1(13.4)	24.8(3.5)	24.8(3.9)	61	169	3.7(2.5)	3.4(2.2)	35/26	81/88	Retroperitoneal
Lairmore	2016	USA	56.5	52.9	28.5	31.23	17	72	2.4(1.3)	2.38(1.2)	11-Jun	40/36	Retroperitoneal
Dickson	2013	USA	52(10.3)	52(13)	31.6(6.1)	30(6)	23	23	3.8(1.6)	2.8(1.2)	13/10	11-Dec	Retroperitoneal
Agcaoglu	2012	USA	52.5(12.81)	53.2(11.14)	27.5(3.9)	30.3(4.45)	31	31	3.1(1.11)	3(1.11)	16/15	19-Dec	Retroperitoneal

RPRA, robotic posterior retroperitoneal adrenalectomy; LPRA, laparoscopic posterior retroperitoneal adrenalectomy; Mean (SD).

**Table 2:** Surgical outcomes.

Reference	operative time(mins)		blood loss (ml)		hospital stay (days)		conversion (n)		transfusion (n)		complications (n)	
	RPRA	LPRA	RPRA	LPRA	RPRA	LPRA	RPRA	LPRA	RPRA	LPRA	RPRA	LPRA
Isiktas	170.6(80.7)	188(101.85)	NA		1.1(0.5)	3.1(0.5)	0	0	NA		overall:0	overall:1
Ma	163(56.23)	165.7(52.89)	50(59.26)	50(66.67)	3(0.74)	4(1.48)	0	0	0	2	major:0	overall:2 major:0
Fu	166.3(54.0)	165(69.5)	100(111.1)	200(162.96))	5(0.74)	6(1.48)	NA	NA	1	7	overall:6 major:1	overall:9 major:2
Kim	138(54.5)	110(50.9)	NA		NA		0	0	NA		NA	
Lairmore	177.3(50.1)	105.33(29.60)	46.5(25.4)	78.4(141.5)	1.53(0.87)	1.85(1.5)	0	3	0	1	major:1	overall:1 major:1
Dickson	154(43)	131(41)	28.3(50.9)	20(37.4)	1.3(0.6)	1.4(0.7)	NA	NA	NA		NA	
Agcaoglu	163(56.23)	165.7(52.89)	25.3(57.35)	35.6(55.12)	NA	NA	0	0	NA		major:0	major:0

RPRA, robotic posterior retroperitoneal adrenalectomy; LPRA, laparoscopic posterior retroperitoneal adrenalectomy; Mean (SD).

**Table 3:** Oncologic outcomes.

Reference	RPRA	LPRA
Isiktas	Pheochromocytoma:3; Aldosteronism:5 Cushing syndrome:4; Other:3	Pheochromocytoma:3; Aldosteronism:5 Cushing syndrome:12; Other:4
Ma	Pheochromocytoma:19; Aldosteronism:5 Cushing syndrome:25; Other:30	Pheochromocytoma:18; Aldosteronism:5 Cushing syndrome:25; Other:31
Fu	Pheochromocytoma:19	Pheochromocytoma:32
Kim	Pheochromocytoma:24; Aldosteronism:9 Cushing syndrome:22; Other:6	Pheochromocytoma:54; Aldosteronism:42 Cushing syndrome:47; Other:26
Lairmore	Pheochromocytoma:20; Aldosteronism:23; Cushing syndrome:20; Other:26	
Dickson	Pheochromocytoma:8; Aldosteronism:3 Cushing syndrome:8; Other:4	NA
Agcaoglu	Pheochromocytoma:6; Aldosteronism:6 Cushing syndrome:13; Other:6	Pheochromocytoma:7; Aldosteronism:8 Cushing syndrome:13; Other:3

RPRA, robotic posterior retroperitoneal adrenalectomy; LPRA, laparoscopic posterior retroperitoneal adrenalectomy.

### 3.2 Assessment of quality

No randomized controlled trials (RCTs) comparing RPRA to LPRA were identified. The current meta-analysis meticulously scrutinized a cumulative of seven meticulously chosen investigations, with six of these displaying a discernible, moderate proclivity for bias, while only a study exhibited a notably diminished susceptibility to bias (14). Notably, each of the incorporated studies executed a meticulous comparative scrutiny, as elucidated in **Table S1**.

### 3.3 Outcome analysis

#### 3.3.1 Perioperative effectiveness

**Figure 2:**
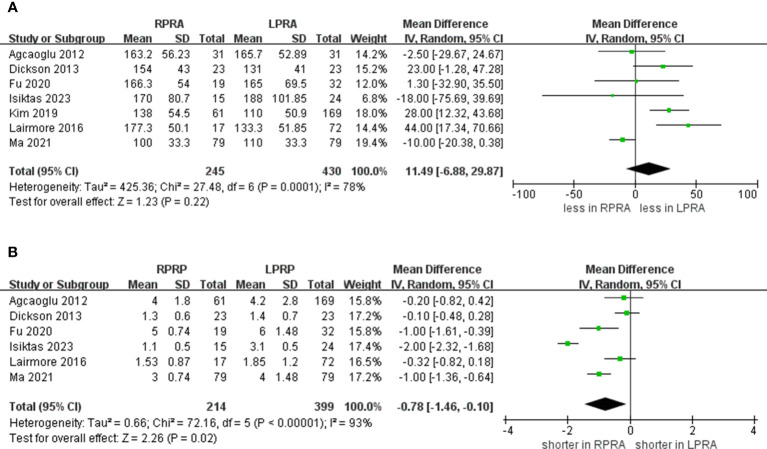
Forest plots of perioperative outcomes for RPRA versus LPRA. **(A)** operative time, **(B)** length of hospital stay.

Following the amalgamation of findings from seven studies, no statistically significant distinction emerged in operative time between the RPRA and LPRA approaches (p = 0.22) (14–20). Upon pooling data from six distinct studies, the RPRA cohort exhibited a diminished duration of hospitalization compared to their LPRA counterparts (WMD -0.78 days, 95% CI -1.46 to -0.10; p = 0.02) (14–16, 18–20) (**Figure 2**).

**Figure 3:**
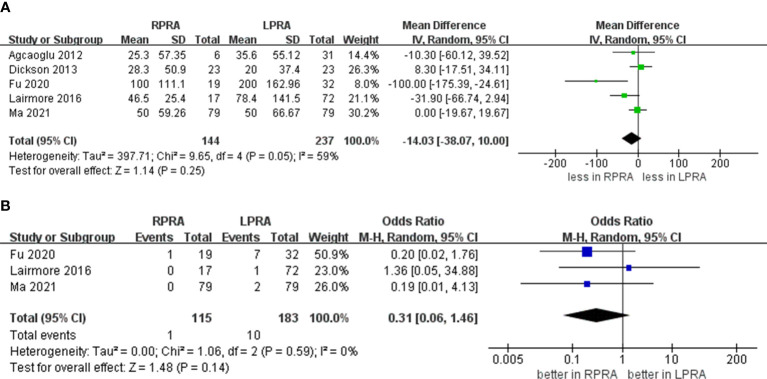
Forest plots of perioperative outcomes for RPRA versus LPRA. **(A)** blood loss, **(B)** transfusion rates.

**Figure 4:**
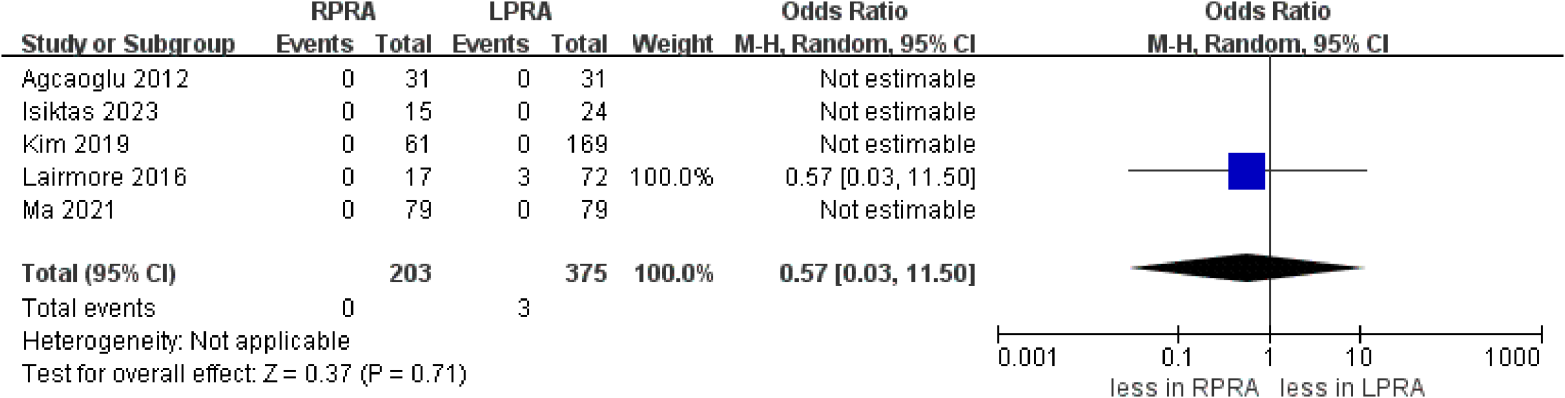
Forest plots of perioperative outcomes for RPRA versus LPRA. Conversion to open.

The cumulative revealed no statistically significant disparity in the occurrence of blood loss (five studies; p = 0.25) (15, 16, 18–20). Likewise, no substantial difference emerged in the prevalence of transfusion rate between RPRA and LPRA (p = 0.14, three studies) (**Figure 3**) (15, 16, 18). The frequency of conversion to open surgery was documented in five studies. However, the aggregated analysis did not reveal any statistically significant disparities in the reduction of conversion to open surgery between RPRA and LPRA (p = 0.71) (**Figure 4**) (14, 15, 17, 18, 20).

#### 3.3.2 Complications

**Figure 5:**
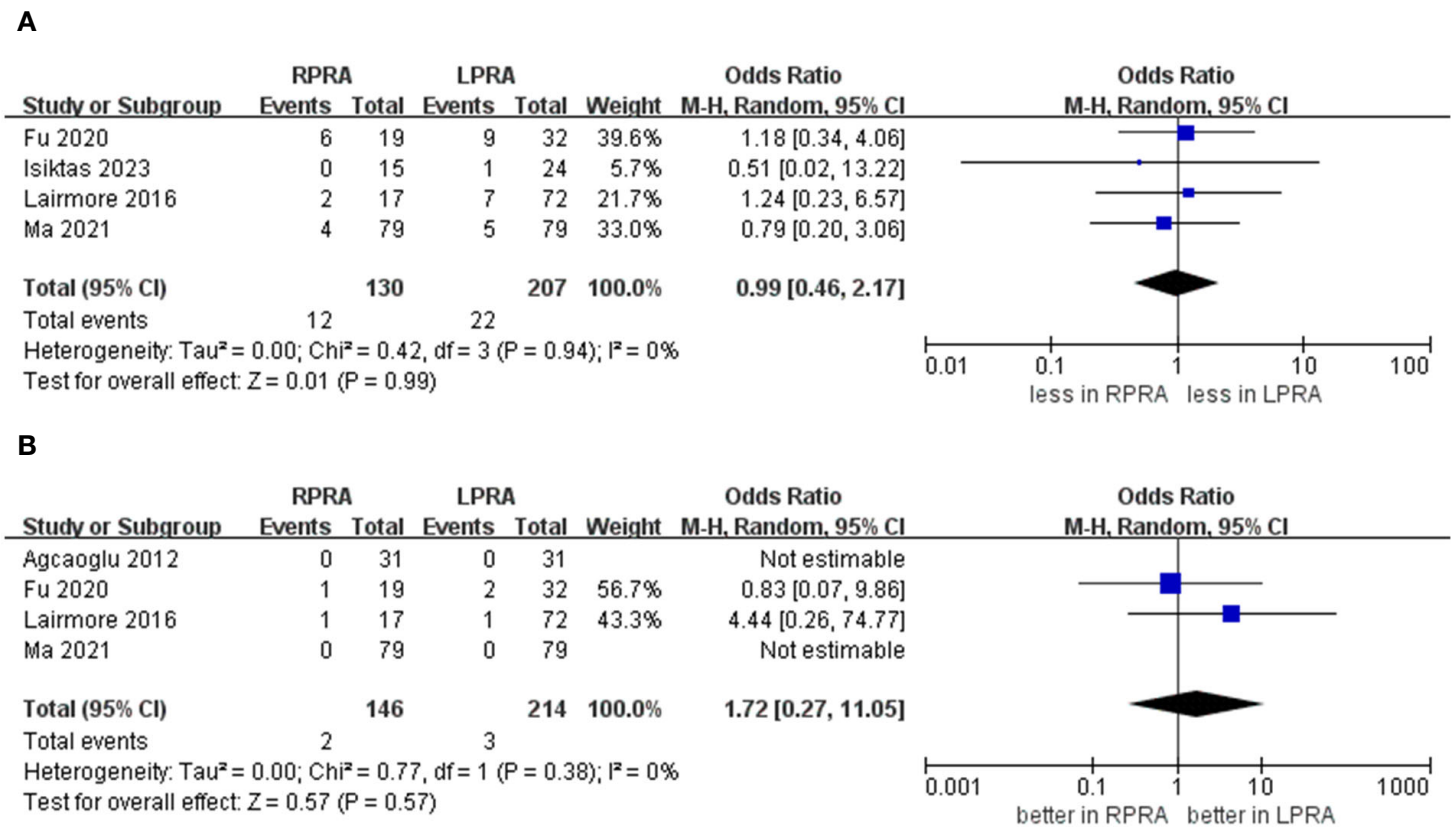
Forest plots of complication for MIPN versus OPN. **(A)** overall complication, **(B)** major complications.

The collective incidence of overall complications was 9.2% (12 out of 130 cases) for RPRA and 10.6% (22 of 207 cases) for LPRA (14–16, 18). Notably, no substantial disparities emerged in the prevalence of postoperative overall complications (graded as Clavien ≥1) (p = 0.99). Moreover, the rates of major complications were 1.3% (2 out of 146 cases) for RPRA and 1.4% (3 of 214 cases) for LPRA. Similarly, no statistically significant differences were identified in the occurrence of major complications between RPRA and LPRA (four studies; p = 0.57) (**Figure 5**) (15–18).

#### 3.3.3 Subgroup

We undertook a subgroup analysis through the stratification of data according to age, BMI, sample size, and tumor diameter. This rigorous analysis encompassed pivotal outcomes, including operative time, length of hospitalization, and blood loss, all of which are presented in **Table 4**.

**Table 4:** Subgroup analysis of perioperative and oncologic outcomes for RPRA and LPRA.

Group	Subgroups	Studies (n)	MD/OR (95% CI)	I^2^ (%)	*P*
Operative time					
Age	Mean age < 50 years	3	6.58 (-21.73, 34.90)	87	0.65
	Mean age ≥ 50 years	4	16.65 (-7.60, 40.89)	60	0.18
BMI	BMI < 30 kg/m2	5	11.90 (-10.44, 34.24)	84	0.3
	BMI ≥ 30 kg/m2	2	11.19 (-25.19, 47.58)	39	0.55
Sample size	Sample size < 80	4	7.45 (-7.97, 22.87)	0	0.34
	Sample size ≥ 80	3	19.17 (-13.84, 52.18)	92	0.26
Tumor diameter	Tumor diameter < 5 cm	5	12.85 (-7.74, 33.43)	82	0.22
	Tumor diameter ≥ 5 cm	1	-1.30 (-32.90, 35.50)	0	0.94
Length of stay					
Age	Mean age < 50 years	2	-1.00 (-1.31, -0.69)	0	< 0.00001
	Mean age ≥ 50 years	4	-0.67 (-1.72, 0.39)	96	0.22
BMI	BMI < 30 kg/m2	4	-0.65 (-1.08, -0.23)	64	0.003
	BMI ≥ 30 kg/m2	2	-0.78 (-2.15, 0.60)	97	0.27
Sample size	Sample size < 80	4	-0.83 (-1.89, 0.22)	95	0.12
	Sample size ≥ 80	2	-0.68 (-1.35, -0.02)	79	0.04
Tumor diameter	Tumor diameter < 5 cm	5	-0.74 (-1.52, 0.05)	94	0.07
	Tumor diameter ≥ 5 cm	1	-1.00 (-1.61, -0.39)	0	0.001
Blood loss					
Age	Mean age < 50 years	2	-43.11 (-140.17, 53.96)	84	0.38
	Mean age ≥ 50 years	3	-9.03 (-35.22, 17.17)	40	0.5
BMI	BMI < 30 kg/m2	4	-24.08 (-56.07, 7.92)	62	0.14
	BMI ≥ 30 kg/m2	1	8.30 (-17.51, 34.11)	0	0.53
Sample size	Sample size < 80	3	-23.82 (-76.05, 28.41)	72	0.37
	Sample size ≥ 80	2	-12.58 (-43.13, 17.98)	59	0.42
Tumor diameter	Tumor diameter < 5 cm	4	-4.11 (-19.62, 11.40)	15	0.6
	Tumor diameter ≥ 5 cm	1	-100.00 (-175.39, -24.61)	0	0.009

All subgroup analyses consistently indicated no significant disparity in operative time between the two groups. The heterogeneity across studies concerning length of hospital stay was found to be influenced by both age and tumor diameter. Specifically, within the subset of studies involving individuals aged < 50 years, RPRA exhibited a markedly reduced length of stay compared to LPRA (p < 0.00001). In contrast, within the subgroup of studies encompassing individuals aged ≥ 50 years, no significant variance in length of stay was observed between the two groups (p = 0.22). Furthermore, within the subgroup characterized by a tumor diameter ≥ 5 cm, patients who underwent RPRA displayed a significantly shorter length of hospital stay compared to those who underwent LPRA (p = 0.001). Conversely, no noteworthy distinction was noted within the subgroup of cases with a tumor diameter < 5 cm (p = 0.07).

Our analysis revealed tumor diameter as a substantive source of heterogeneity concerning blood loss. In particular, within the subset characterized by a tumor diameter ≥ 5 cm, RPRA exhibited an association with diminished blood loss in contrast to LPRA (p = 0.009), whereas in the subgroup marked by a mean tumor diameter < 5 cm, no noteworthy distinction was evident (p = 0.6).

### 3.4 Heterogeneity

A prevailing trend toward low to moderate heterogeneity was evident in most of the findings. Even with the incorporation of studies of intermediate and high quality, substantial variability was discerned in two of the outcomes (operative time, I^2^ = 78%; length of hospital stay, I^2^ = 93%).

### 3.5 Sensitivity analysis

Within the context of this study, the evident heterogeneity present in factors such as operative time and hospital stay prompted the implementation of a sensitivity analysis. This analytical endeavor aimed to unveil the fundamental origins of the heterogeneity while also evaluating the robustness and steadfastness of the study’s outcomes. The findings of this comprehensive analysis unveiled a lack of substantial shifts in the extent of heterogeneity, signifying the enduring consistency of the underlying heterogeneity sources in both operative time and hospital stay over the course of the study.

### 3.6 Publication bias

**Figure 6:**
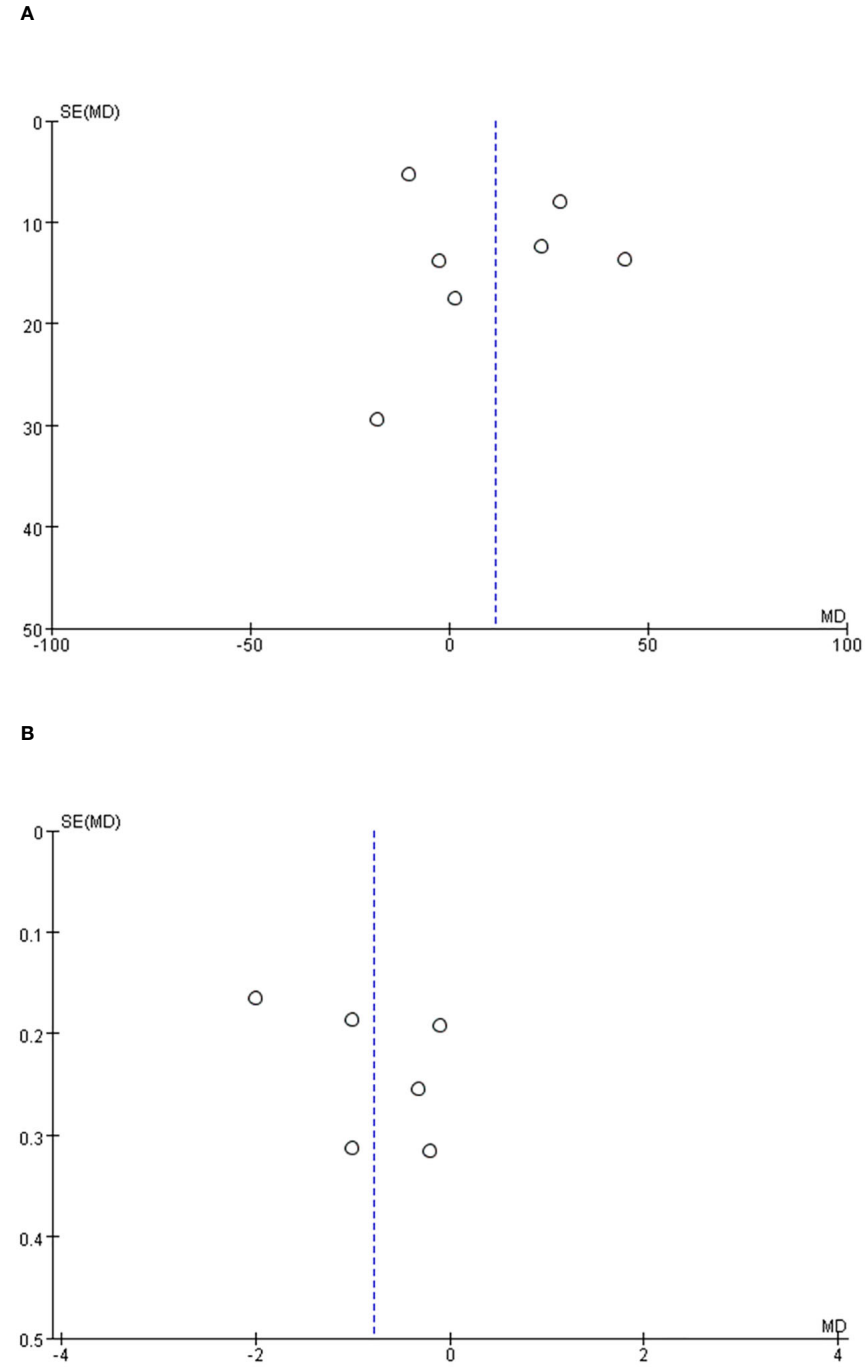
Funnel plot of the studies represented in the meta-analysis. **(A)** operative time, **(B)** length of hospital stay.

To ascertain the potential for publication bias within the examined studies, an analysis was conducted involving operative time, and length of stay as variables. Our findings revealed that the distribution among the studies exhibited an almost symmetrical pattern, suggesting a minimal probability of publication bias (**Figure 6**).

## 4 Discussion

This represents the first systematic review and meta-analysis examining the comparative outcomes between RPRA and LPRA. Several pivotal discoveries within this study merit comprehensive elucidation and discourse.

Seven studies were encompassed in the analysis of operative duration. No statistically significant difference was observed in operative time between RPRA and LPRA. Nevertheless, earlier investigations revealed a substantial elongation in the procedural duration for robotic-assisted posterior retroperitoneoscopic adrenalectomy in contrast to its posterior retroperitoneoscopic counterpart (21, 22). After establishing pneumoperitoneum, three to four robotic ports are typically positioned two finger-widths below the rib edge. Additionally, there are instances where it becomes necessary to create an initial auxiliary opening near the border of the rectus muscle to facilitate retraction or suction (23, 24). The surgeon’s preparatory actions, encompassing the orchestration of the operative field, calibration of camera perspectives, and manipulative proficiency, may have exerted influence on the temporal course of RPRA procedures. Furthermore, the variable levels of surgical expertise possessed by individual practitioners bore impact on the operative time within the aggregate studies. In light of recent investigations, as surgeons traverse the learning curve, the temporal demands associated with the robotic approach are anticipated to diminish. In addition, the favorable outcomes observed in robotic urology surgeries conducted by RPRA surgeons may be ascribed to their expertise gained through previous experience in other robotic procedures, such as robotic prostatectomy and nephrectomy (1). An explicable conjecture could attribute this phenomenon to the prevalence of more contemporary RPRA interventions, a manifestation likely stemming from the evolutionary aspects of the surgical technique. Hence, it is conceivable that the surgeon’s navigation through the learning curve could inadvertently protract the operative duration (8, 25). Indeed, within the recent inclusions, RPRA has demonstrated a notably reduced operative time in comparison to LPRA. The integration of robotic articulatory instruments with a more robust camera platform and the provision of high-definition 3D visualizations have the potential to expedite the dissection process. Therefore, it remains conceivable that RPRA may have the potential to necessitate a reduced operative duration compared to LARP in subsequent periods (26, 27). Accordingly, a more substantial body of high-caliber evidence is imperative to substantiate our findings. No statistically significant disparity surfaced in the conversion rate between RPRA and LARP. A prior investigation documented a RPRA conversion rate reaching a magnitude of 40%, thereby unveiling an elevated conversion propensity within the RPRA domain as juxtaposed with LARP (28). Concomitant with the accumulated proficiency of individual surgeons utilizing the robotic platform, the conversion rates exhibited a parallel reduction akin to the corresponding levels observed within the LARP frame (29).

Notwithstanding the divergent findings reported in antecedent studies regarding hospital stay (20, 30), our conducted meta-analysis tends to corroborate that RPRA was linked to a briefer hospitalization interval in comparison to LPRA. The variance in hospital stay can be elucidated through the subsequent rationales. Primarily, this variance could potentially stem from the advantages intrinsic to the robotic platform. The benefits conferred by robotic technology encompass high-resolution three-dimensional optics, augmented dexterity, and improved ergonomics, enabling quiver-free and meticulous movements (31). Additionally, the sensitivity analysis indicates the robustness of the estimations. Secondly, bearing in mind the consistent demonstration in prior research of the pivotal role played by institutional caseload and surgeon expertise as crucial determinants influencing the outcomes of minimally invasive procedures, RPRA and LARP are not exempt from this paradigm (32). Hence, exercising prudence is imperative while appraising the hospitalization period following RPRA and LPRA.

Blood loss stands as a pivotal metric for assessing surgical quality. While a statistically significant discrepancy in blood loss between the two groups was not observed, the majority of the encompassed studies exhibited that the RPRA cohort manifested a diminished transfusion incidence and reduced blood loss in contrast to the LARP cohort. The variance in estimated blood loss can be explicated through the ensuing rationales. The adaptability of the robotic flexible arm coupled with the enhanced precision afforded by magnified high-definition stereoscopic vision facilitates the identification and management of intraoperative bleeding and the precise delineation of intricate anatomical structures and separations (33). However, what is worth our attention is that estimate blood loss is not a relevant parameter to assess the surgical efficacy, because of a difference of few ml may not be clinically significant. Furthermore, it is essential to consider that the postoperative blood transfusion rate among patients may be contingent on the surgical expertise of the healthcare professionals involved and the specific blood transfusion protocols adhered to by the hospitals (34). In forthcoming times, a deeper reservoir of research focusing on blood loss is requisite to further corroborate this assertion.

Regarding morbidity, no noteworthy distinction emerged between RPRA and LARP in relation to both overall and major complications. A precedent meta-analysis substantiated that RPRA exhibited a higher incidence of complications in contrast to LPRA (35). Nonetheless, contemporary investigations have demonstrated a lack of significant divergence between the two groups concerning complication rates (36). The variance in complication rates can be elucidated through the subsequent rationales. Primarily, the accumulated surgeon expertise in robotic utilization has contributed to the reduction in RPRA-associated complications. Secondly, the abbreviated hospital stay and mitigated blood loss appear to equilibrate the physiological strain endured by the surgical patient, thereby culminating in commensurate complication rates.

Given that the elevated cost associated with robotic surgery constitutes a drawback of the procedure, cost assumes a pivotal role in the contemplation of robotic utilization. Several studies have indicated that robotic surgery has been documented to be 1.3 -3.2 times pricier compared to laparoscopy (28, 37). On one hand, the depreciation of the robotic system and augmenting the annual volume of robotic cases employed proved more efficacious in cost mitigation. On the other hand, patients’ selection of an approach was influenced by their individual financial capacity. Hence, cost may not be deemed a salient determinant impacting outcomes. Nevertheless, it exerted influence on infrastructure and medical insurance provisions. It is incumbent upon us to deliberate upon which patients are suitable candidates for the robotic approach, taking into account the social and economic costs.

A subgroup analysis was conducted to ascertain the patient cohort that could potentially derive advantages from RPRA as opposed to LPRA. Certain studies have posited that LTA for tumors surpassing the 5 cm threshold is both secure and feasible under the supervision of a seasoned practitioner. Despite the anticipation of lengthier operative durations associated with LPRA in comparison to LTA, select research endeavors have indicated that the employment of a robotic platform could potentially truncate the procedural chronometry for adrenal tumors > 5 cm (9, 38). Nevertheless, our study did not disclose statistically significant disparities in operative time. Despite the subgroup analysis unveiling a shorter postoperative hospitalization duration within the RPRA cohort, no marked distinctions between the two surgical methodologies were discerned in relation to other outcomes. This could potentially be attributed to the diminutive sample size. Given the inherent advantages of robotic platforms, some surgeons may opt for robotic methods to manage more challenging cases, such as larger tumors or patients with higher BMI (39). Consequently, additional investigations are imperative to facilitate a comprehensive comparison of the advantages intrinsic to both approaches within these cases. Upon stimulation, pheochromocytomas can exude catecholamines, precipitating hemodynamic fluctuations that may culminate in severe complications or morbidity. Consequently, adrenalectomy for pheochromocytoma presents a substantial challenge for operators. In recent years, several studies have postulated that the robotic platform confers superior therapeutic efficacy in the context of adrenal tumors with pheochromocytoma (40, 41). Particularly for pheochromocytomas, neoplasms characterized by a profuse vascular supply, precision in surgical execution holds paramount significance for efficacious hemostatic management. With the exception of operative time, no significant distinctions were observed between the two groups. In light of the paucity of data within our study, circumspection is warranted while appraising the outcomes differential between RPRA and LPRA in the context of pheochromocytoma. Furthermore, Li et al. (42) conducted a meticulous meta-analysis to comprehensively assess the safety and efficacy of partial adrenalectomy (PA) in comparison to total adrenalectomy (TA), with a focus on perioperative and functional outcomes. Their analysis reveals that surgical outcomes in both TA and PA procedures are indeed comparable. The robotic system appears exceptionally well-suited for this technology, owing to its remarkable capacity for achieving precise operations. This is primarily attributed to its multi-degree-of-freedom articulated wrist and the advantage of 3D magnified vision (24). However, further research is imperative to corroborate this conclusion. We have added these relevant findings in the current manuscript.

The current study bears certain limitations that necessitate acknowledgment prior to the interpretation of our findings. First and foremost, the composition of non-randomized controlled trials (non-RCTs) with intermediate methodological quality engenders susceptibility to potential misclassification bias and latent confounding variables. Moreover, a subset of the incorporated studies exhibited diminutive sample sizes. Secondly, albeit the utilization of subgroup analysis, it is noteworthy that the amalgamated studies encompass diverse clinical diagnoses for tumors, a factor that potentially introduces confounding elements into the results. Thirdly, the preponderance of outcomes is contingent upon a selection of the seven studies, precipitated by a dearth of data within the remaining studies. This aspect conspicuously underscores the limitations inherent in the comparison. A substantial portion of the studies are characterized by limited sample sizes and inadequate statistical power.

## 5 Conclusions

The outcomes of this meta-analysis provide encouragement for the adoption of RPRA within the retroperitoneal space. Specifically, RPRA exhibited a reduced hospitalization duration and diminished invasiveness. Furthermore, the study indicates comparable perioperative outcomes and complication rates when juxtaposed with LPRA. Given that the encompassed studies were characterized by non-randomized controlled trials (non-RCTs) with intermediate methodological quality, the substantiation of RPRA’s superiority and identification of the patients most predisposed to gain from RPRA mandate the execution of prospective randomized controlled trials (RCTs) with extended follow-up periods and elevated-level evidence.

## Data availability statement

The original contributions presented in the study are included in the article/**Supplementary Material**. Further inquiries can be directed to the corresponding author.

## Author contributions

Y-GL: Conceptualization, Data curation, Formal analysis, Funding acquisition, Investigation, Methodology, Project administration, Resources, Software, Supervision, Validation, Visualization, Writing – original draft, Writing – review & editing. X-BC: Conceptualization, Formal analysis, Resources, Visualization, Writing – original draft, Writing – review & editing. C-MW: Conceptualization, Data curation, Formal analysis, Project administration, Supervision, Validation, Writing – original draft, Writing – review & editing. X-DY: Formal analysis, Investigation, Visualization, Writing – original draft, Writing – review & editing. X-ZD: Conceptualization, Formal analysis, Writing – original draft, Writing – review & editing. BL: Conceptualization, Data curation, Formal analysis, Funding acquisition, Methodology, Writing – original draft, Writing – review & editing.
