# Supplementary material for: Robotic posterior retroperitoneal adrenalectomy versus laparoscopic posterior retroperitoneal adrenalectomy: outcomes from a pooled analysis
Source: Front Endocrinol (Lausanne). 2023 Nov 28;14:1278007. doi: 10.3389/fendo.2023.1278007 (PMC10715275; doi:10.3389/fendo.2023.1278007)
Supplement: Supplementary file 1 [file Table_1.docx]

| **Table S1 The risk of bias (Non-RCTs)-ROBINS-I** | | | | | | |  |  |
| --- | --- | --- | --- | --- | --- | --- | --- | --- |
| Bias domain | Isiktas | Ma | Fu | Kim | Lairmore | Dickson | Agcaoglu |  |
|  |  |  |  |  |  |  |  |  |
| Bias due to confounding | Moderate | Moderate | Moderate | Moderate | Moderate | Moderate | Moderate |  |
|  |  |  |  |  |  |  |  |  |
| Bias in selection of participants into the study | Low | Low | Low | Low | Low | Low | Low |  |
|  |  |  |  |  |  |  |  |  |
| Bias in classification of interventions | Low | Low | Low | Low | Low | Low | Low |  |
|  |  |  |  |  |  |  |  |  |
| Bias due to deviations from intended interventions | Low | Low | Low | Moderate | Moderate | Low | Moderate |  |
|  |  |  |  |  |  |  |  |  |
| Bias due to missing data | Low | Moderate | Moderate | Low | Low | Moderate | Low |  |
|  |  |  |  |  |  |  |  |  |
| Bias in measurement of outcomes | Low | Low | Low | Moderate | Low | Moderate | Low |  |
|  |  |  |  |  |  |  |  |  |
| Bias in selection of the reported result | Moderate | Moderate | Moderate | Moderate | Moderate | Moderate | Moderate |  |
|  |  |  |  |  |  |  |  |  |
| Overall bias | Low | Moderate | Moderate | Moderate | Moderate | Moderate | Moderate |  |
|  |  |  |  |  |  |  |  |  |
|  |  |  |  |  |  |  |  |  |
